# Dose- and endpoint-dependent effects of perioperative esketamine in children: evidence from a targeted review

**DOI:** 10.3389/fped.2026.1963881

**Published:** 2026-10-21

**Authors:** Yun Yang, Tongyun He, Weiliang Mao, Xinqiang Wang

**Affiliations:** The First People’s Hospital of Huzhou, Huzhou, China

**Keywords:** dexmedetomidine, dose–response relationship, drug safety, emergence delirium, esketamine, paediatric anaesthesia, pharmacokinetics

## Abstract

Esketamine is being evaluated for several paediatric perioperative indications, including analgesia, procedural sedation, attenuation of propofol injection pain, and prevention of emergence delirium (ED) or emergence agitation (EA). This mini review examines whether evidence identified in a targeted search supports a clinically useful therapeutic window and how dose, timing, route, developmental stage, anaesthetic technique, procedure, co-medication, and outcome definition modify treatment effects. Randomised trials and meta-analyses generally report fewer ED or EA events after low intravenous doses, particularly during sevoflurane anaesthesia for adenotonsillectomy. Direct dose-comparison evidence shows increasing benefit up to 0.5 mg·kg^−1^ intravenously in that setting, without identifying an upper efficacy or safety boundary. An observational association between esketamine exposure and more ED cannot establish a threshold because treatment selection and perioperative context were not controlled. Effective doses are endpoint-specific: propofol injection pain, procedural sedation, analgesia, and emergence outcomes require different exposure profiles. Intranasal regimens cannot be converted into intravenous equivalents from bioavailability alone, and preclinical neurotoxicity findings do not define a ceiling for a single perioperative dose in children. Among the studies identified, 0.2–0.5 mg·kg^−1^ was the best-studied intravenous range in selected emergence-prevention settings, not a universal recommendation or validated therapeutic window. Future trials should compare dose and timing directly, incorporate paediatric pharmacokinetic–pharmacodynamic sampling, use endpoint-specific assessment, and extend safety follow-up.

## 1 Introduction

Esketamine, the S-enantiomer of ketamine, has greater affinity for the N-methyl-D-aspartate receptor than racemic ketamine (1). In children, it is increasingly used as an adjunct for analgesia, procedural sedation, attenuation of propofol injection pain, and prevention of emergence agitation (EA) or emergence delirium (ED). However, most paediatric perioperative indications remain dependent on local regulatory approval and practice, and the evidence is concentrated in single-centre studies involving a limited range of procedures.

The literature appears discordant. A prospective observational study of 230 preschool children associated esketamine exposure (mean dose, 0.46 mg·kg^−1^) with more ED and a longer post-anaesthesia care unit stay (2), whereas subsequent randomised trials and meta-analyses generally reported benefit (3–6). These findings cannot be reconciled by dose alone because treatment allocation, procedure, timing, anaesthetic technique, baseline anxiety, and outcome definitions differed. The clinically relevant question is whether evidence identified in the targeted search defines indication-specific exposure ranges with an acceptable balance of benefit and harm. Figure 1 summarises this framework and distinguishes the studied low-dose range from the uncertainties that prevent definition of a universal therapeutic window.

**Figure 1:**
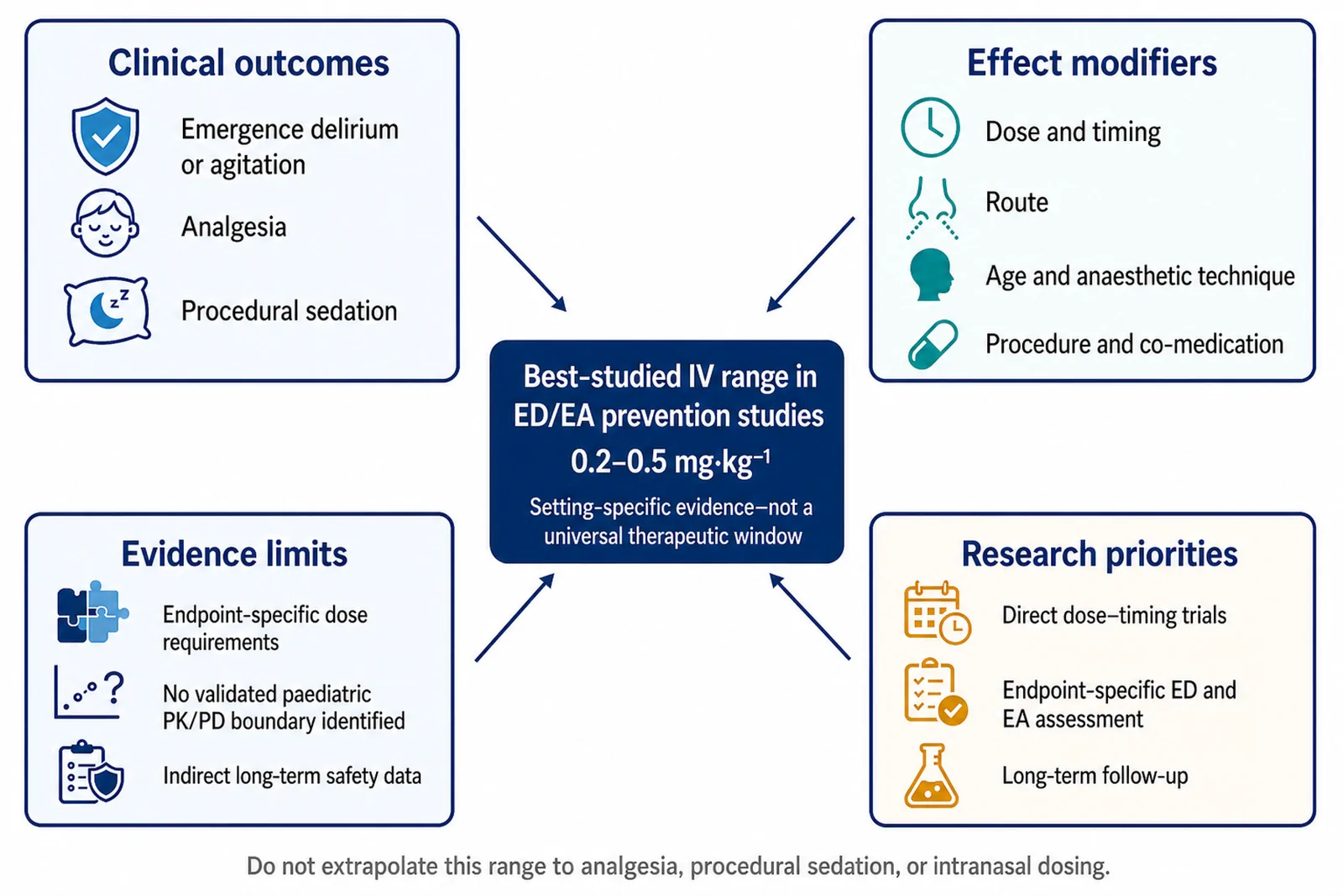
Evidence framework for perioperative esketamine in children. Intravenous doses of 0.2–0.5 mg·kg^−1^ are the best-studied range in trials assessing emergence delirium or agitation, principally during sevoflurane anaesthesia for adenotonsillectomy. The terms retain the definitions used in each source study and do not constitute a standardised composite endpoint. This range should not be extrapolated to analgesia, procedural sedation, intranasal administration, or other procedures. ED, emergence delirium; EA, emergence agitation; PK/PD, pharmacokinetics/pharmacodynamics.

In this review, “therapeutic window” is used as a conceptual framework rather than a formally established concentration range. A studied dose range denotes doses evaluated in trials; an effective dose range requires reproducible benefit for a specified endpoint; an exposure range refers to measured systemic drug exposure; and a therapeutic window requires both a lower efficacy boundary and an upper toxicity boundary. These terms are not interchangeable. Because paediatric concentration–effect and concentration–toxicity data are unavailable, the present analysis can identify only context-specific studied or effective dose ranges.

Recent systematic reviews have estimated average effects of perioperative esketamine on ED or EA (3, 4, 7). This mini review addresses a different question: it evaluates whether dose, route, timing, and endpoint-specific evidence can define lower and upper boundaries. It therefore separates injection pain, procedural sedation, analgesia, and emergence outcomes; examines developmental PK/PD uncertainty; and considers whether combination regimens or available safety data change the interpretation of those boundaries.

## 2 Methods

We conducted a targeted narrative search of PubMed/MEDLINE and the reference lists of relevant trials and reviews for publications from 1 January 2006 to 10 August 2026. The complete Boolean strategy is provided in Supplementary Table S1. The PubMed search identified 166 records. After title and abstract screening, reference-list checking, and exclusion of records outside the focused review question, 14 peer-reviewed paediatric original studies and five directly relevant systematic reviews or meta-analyses were included in the narrative synthesis. Two authors independently screened the records, and disagreements were resolved through discussion. We did not perform a formal risk-of-bias or certainty assessment. We also did not search trial registries; unpublished studies, conference abstracts without a full article, and ongoing trials without peer-reviewed results were excluded. Earlier publications were retained when they provided foundational pharmacology, validated outcome instruments, or landmark neurodevelopmental evidence. Adult PK and animal studies were used only when direct paediatric evidence was unavailable. Embase, Web of Science, Scopus, the Cochrane Library, and non-English databases were not searched, and no quantitative synthesis was performed. The conclusions therefore apply to evidence identified by this targeted strategy rather than to all available literature.

## 3 Why treatment effects differ across studies

### 3.1 Dose, timing, and exposure profile

Paediatric trials have evaluated intravenous boluses of approximately 0.1–1.0 mg·kg^−1^, but few directly compared doses. Administration at induction, during surgery, or near wound closure produces different concentrations at emergence. Meta-analytic subgroup findings favouring a bolus over continuous infusion are indirect and do not establish that ED is determined by peak concentration rather than total exposure (4). We identified no paediatric concentration–effect study that tested this hypothesis.

### 3.2 Anaesthetic technique, procedure, and baseline risk

Sevoflurane exposure, preschool age, preoperative anxiety, and postoperative pain are established correlates of ED (8–12). An adjunct with analgesic and sedative effects may therefore produce a larger absolute benefit when baseline risk is high, as in adenotonsillectomy under sevoflurane. Findings from endoscopy, bronchoscopy, strabismus surgery, hypospadias repair, and neurosurgery should not be treated as estimates of one common effect.

### 3.3 Outcome definition

ED and EA overlap but are not interchangeable. The Pediatric Anesthesia Emergence Delirium (PAED) scale was developed to assess delirium, whereas the Watcha and Cravero scales place greater weight on agitation (13–15). Pain, fear, residual sedation, and dissociative behaviour can influence these assessments. Variation in thresholds and assessment times consequently limits comparisons of incidence and treatment effect.

### 3.4 Developmental pharmacology

Developmental variation in N-methyl-D-aspartate receptor expression provides biological plausibility for age-dependent effects (16), but it does not establish different receptor occupancy or dose thresholds between age groups. Body composition and maturation of hepatic metabolic capacity may alter distribution, clearance, and formation of active metabolites such as noresketamine. Route also matters: intranasal absorption is variable and adds swallowed drug and first-pass metabolism, whereas an intravenous bolus produces a more immediate peak. None of these mechanisms has been quantified in a paediatric perioperative esketamine population model. Apparent age effects may instead reflect baseline ED risk, communication ability, vascular access, and procedure mix. Racemic ketamine models and adult esketamine data can generate hypotheses but cannot justify empirical age-based dose escalation or reduction.

## 4 Clinical evidence by perioperative endpoint

### 4.1 Propofol injection pain and endoscopic response

Propofol injection pain is common in children (17). Published paediatric studies of gastroscope insertion have identified regimen-specific dose requirements within propofol-based sedation protocols (18, 19). These immediate procedural responses should not guide dosing for ED prevention.

### 4.2 Emergence delirium and agitation

Randomised trials generally support a preventive effect. S-ketamine 0.2 mg·kg^−1^ given at the end of adenotonsillectomy reduced ED without prolonging extubation (6), and 0.2 mg·kg^−1^ administered during surgery produced a similar direction of effect in a larger trial (5). Meta-analyses also report lower ED or EA rates, although pooled effects vary with dose definition, procedure, and included studies (3, 4, 7).

The most informative direct comparison randomised 164 children undergoing adenotonsillectomy to 0, 0.1, 0.3, or 0.5 mg·kg^−1^ before induction (20). EA occurred in 62.5%, 56.8%, 25.6%, and 18.9%, respectively. The 0.5 mg·kg^−1^ group had a longer time to tracheal tube removal, but benefit did not reverse. Thus, the study supports a dose-related reduction in EA up to 0.5 mg·kg^−1^ in this setting while leaving the upper efficacy boundary undefined.

### 4.3 Analgesia and procedural sedation

Trials using a 0.5 mg·kg^−1^ bolus or low-dose infusion have reported lower postoperative pain scores or opioid use (21–23). Intranasal esketamine has also been studied for bronchoscopy, premedication, and procedural sedation (24, 25). These applications require different depths and durations of effect. A dose that attenuates a brief procedural response cannot be interpreted as the lower boundary for sustained analgesia, and a sedative regimen cannot define the upper boundary for ED prophylaxis.

Evidence outside adenotonsillectomy remains limited but covers several distinct settings. Published studies include upper gastrointestinal endoscopy, fibreoptic bronchoscopy, hypospadias repair, laryngeal mask insertion, general paediatric surgery, and neurosurgery (18, 19, 22–24, 26, 27). These studies differ in route, comparator, co-medication, and primary outcome. They support feasibility in selected protocols but do not establish that the dose range studied for adenotonsillectomy transfers to other procedures.

Table 1 summarises representative published studies across endpoints and procedures. It also records short-term safety findings and design limitations. The appraisal is descriptive rather than a formal risk-of-bias assessment.

**Table 1:** Representative published paediatric studies with safety findings and critical appraisal.

Study/design	Setting/timing	Regimen	Endpoint	Reported safety	Critical appraisal
Zheng et al. (19); prospective dose-finding study	Upper gastrointestinal endoscopy; before propofol	IV 0, 0.25, 0.5, or 1 mg·kg^−1^	First-insertion response; propofol ED50/ED95	Short observation; efficacy-sized sample	Sequential allocation and endpoint-specific estimate; no emergence or safety boundary
Chen et al. (6); randomised trial	Adenotonsillectomy; end of anaesthesia	IV 0.2 mg·kg^−1^ vs. saline	Maximum PAED; ED by Aono scale	No prolonged extubation reported	Single centre, one dose, and procedure-specific outcome
Lin et al. (5); randomised trial	Adenotonsillectomy; after adenoid resection	IV 0.2 mg·kg^−1^ vs. saline	ED and 30-day behavioural follow-up	No new major short-term signal reported	Larger sample and follow-up, but one procedure and dose
Jiang et al. (20); four-arm randomised trial	Adenotonsillectomy; before induction	IV 0, 0.1, 0.3, or 0.5 mg·kg^−1^	PAED and Watcha; EA incidence	At 0.5 mg·kg^−1^, tube removal was longer; HR and MAP differed among groups	Direct dose comparison; no concentration data and limited power for uncommon harms
Chen et al. (2); prospective observational study	Minor surgery; induction	IV exposure; mean 0.46 mg·kg^−1^	PAED and PACU duration	More ED and longer PACU stay	Non-random treatment selection and residual confounding limit causal inference
Xu et al. (23); randomised trial	Hypospadias repair; postoperative analgesia	IV perioperative esketamine regimen	Pain and analgesic outcomes	Short-term safety assessed within the trial	Extends evidence beyond airway surgery; one procedure and limited follow-up
Xie et al. (24); randomised trial	Fibreoptic bronchoscopy; premedication	IN esketamine 1.0 mg/kg vs IN dexmedetomidine 1.0 μg/kg vs saline	ED and peri-induction outcomes	Brief perioperative adverse-event surveillance	Separate active-treatment groups; the study did not test combination therapy
Liao et al. (28); three-arm randomised trial	Adenotonsillectomy; premedication	IN DEX 2 μg·kg^−1^, ESK 1 mg·kg^−1^, or DEX 1 μg·kg^−1^ + ESK 0.5 mg·kg^−1^	PAED ≥10; sedation and behaviour	Adverse events were mild and infrequent in the reported sample	Active comparators, seven exclusions, and no formal interaction test

This descriptive appraisal does not replace a formal risk-of-bias assessment. DEX, dexmedetomidine; EA, emergence agitation; ED, emergence delirium; ED50, median effective dose; ED95, dose estimated to be effective in 95% of the target population; ESK, esketamine; HR, heart rate; IN, intranasal; IV, intravenous; MAP, mean arterial pressure; PACU, post-anaesthesia care unit; PAED, Pediatric Anesthesia Emergence Delirium.

## 5 Route of administration and pharmacokinetic uncertainty

Adult studies estimate the absolute bioavailability of intranasal esketamine at approximately 50%–54% (29). Bioavailability alone, however, does not make 1.0 mg·kg^−1^ intranasal equivalent to 0.5 mg·kg^−1^ intravenous. The routes differ in peak concentration, time to peak, absorption variability, swallowed fraction, and metabolite exposure. Adult nasal-spray models were developed in healthy volunteers and adults with depression rather than perioperative children. We identified no validated paediatric perioperative esketamine population PK model in the targeted search. Intranasal and intravenous regimens should therefore be interpreted separately until paediatric exposure–response data are available (25, 26, 29, 30).

## 6 Safety and the unresolved upper boundary

The clinical evidence identified in the targeted search does not define an upper boundary. The observational association reported by Chen et al. (2) is an important safety signal, but it cannot locate a dose–response inflection point because exposure was not randomised and prespecified dose levels were not compared. Preoperative anxiety, clinical selection, and perioperative management may have contributed. Benefit has also been reported with 0.5 mg·kg^−1^ intravenously and 1.0 mg·kg^−1^ intranasally (3, 20, 24). The evidence therefore demonstrates clinical heterogeneity rather than a confirmed reversal between 0.4 and 0.5 mg·kg^−1^.

Psychotomimetic effects are dose-related in adults receiving ketamine (31), but thresholds for adult racemic ketamine cannot be converted by receptor affinity into a paediatric esketamine ceiling. Younger children may not reliably report dissociation, and common ED instruments do not distinguish dissociation from pain, fear, or residual anaesthesia. Future studies should prospectively record dissociative, haemodynamic, respiratory, and recovery outcomes as separate safety domains.

High-dose S-ketamine in neonatal mice and prolonged ketamine exposure in developing non-human primates have produced neurobehavioural or histological injury (32–35). These models differ substantially from a single low-dose perioperative exposure in dose, duration, developmental stage, and surgical stimulation. They justify longer clinical surveillance but cannot define a neurotoxicity threshold near 0.5 mg·kg^−1^ in children. Similarly, the US Food and Drug Administration warning concerns repeated or prolonged exposure to general anaesthetic and sedative drugs in children younger than 3 years; it is not evidence of harm from one low-dose esketamine bolus (36). The GAS trial is reassuring for a single short general anaesthetic but did not evaluate esketamine (37).

### 6.1 Clinically observed adverse effects

Paediatric trials more commonly report short-term outcomes than neurodevelopment. These include changes in heart rate and blood pressure, respiratory events or oxygen desaturation, nausea and vomiting, abnormal behaviour or psychotomimetic phenomena, salivation, recovery time, tracheal tube removal, and PACU stay. In the direct four-dose trial, heart rate and mean arterial pressure differed among groups and 0.5 mg·kg^−1^ prolonged tracheal tube removal, although PACU duration and postoperative complications did not differ significantly (20). The observational study associated exposure with longer PACU stay (2). Comparison across trials is further limited by inconsistent adverse-event definitions, observation periods, and reporting thresholds. Most trials were powered for efficacy rather than uncommon adverse events; a non-significant difference does not establish safety or an upper therapeutic boundary.

## 7 Co-administration with dexmedetomidine

Dexmedetomidine–esketamine combinations are attractive because the drugs have different sedative, analgesic, and haemodynamic profiles (40). In a three-arm trial, intranasal dexmedetomidine 1 μg·kg^−1^ plus esketamine 0.5 mg·kg^−1^ reduced ED compared with esketamine 1 mg·kg^−1^ alone, but not compared with dexmedetomidine 2 μg·kg^−1^ alone (28). The combination improved several peri-induction outcomes, yet the design did not establish pharmacological synergy or show that dexmedetomidine raises an esketamine safety ceiling.

Another trial of intranasal dexmedetomidine 1 μg·kg^−1^ plus esketamine 0.6 mg·kg^−1^ reported less postoperative pain and fewer ED events than saline, while comparisons with dexmedetomidine alone varied by outcome (38). More recent evidence includes a neurosurgical trial comparing intravenous combination therapy with saline (27) and a meta-analysis favouring intranasal combination therapy over dexmedetomidine alone (39). Although encouraging, heterogeneity in route, dose, procedure, and comparators precludes a universal “half-dose” strategy.

Demonstrating pharmacological synergy would require a factorial or response-surface design, prespecified interaction testing, and comparison at equivalent levels of sedation. Current trials do not meet these criteria.

## 8 Discussion

The central finding is that paediatric perioperative evidence supports a context-dependent studied range rather than a universal therapeutic window. Intravenous doses of 0.2–0.5 mg·kg^−1^ are the best studied for prevention of emergence outcomes, particularly during sevoflurane anaesthesia for adenotonsillectomy. This interval is not a recommended range for every child, procedure, or indication. Its apparent lower boundary depends on the intended clinical endpoint, and its upper boundary remains undefined because few trials directly compare dose levels while measuring both benefit and harm.

This interpretation complements, but is not equivalent to, recent meta-analyses. Those reviews generally found fewer ED or EA events after esketamine but also reported substantial variation in dose, administration strategy, comparator, and assessment instrument (3, 4, 7). Their pooled estimates answer whether esketamine has an average preventive effect across included studies. They do not establish that ED and EA are interchangeable, identify a concentration threshold, or determine whether 0.2–0.5 mg·kg^−1^ applies beyond the emergence settings that dominate the evidence. The present endpoint- and route-specific synthesis is intended to prevent that extrapolation.

### 8.1 Quality and certainty of the evidence

Most paediatric evidence comes from single-centre randomised trials with modest samples and short follow-up. Procedures, anaesthetic protocols, doses, administration times, comparators, and outcome instruments vary substantially. Few trials compare multiple esketamine doses directly, and most were designed to detect efficacy rather than uncommon adverse events. The observational study is vulnerable to confounding by indication and treatment selection (2). These limitations reduce confidence in a universal dose boundary even when individual trials report benefit.

The systematic reviews broaden the evidence base but inherit the clinical and methodological heterogeneity of their included trials (3, 4, 7). Publication bias and small-study effects cannot be excluded. Because this targeted review did not perform a formal risk-of-bias assessment or GRADE evaluation, it does not assign certainty ratings. Taken together, the evidence is sufficient to describe studied ranges and endpoint-specific signals, but not to define a validated therapeutic window or a safety ceiling. Table 1 provides a structured descriptive appraisal of representative studies.

### 8.2 Clinical implications

Dose selection should begin with the intended endpoint, route, timing, anaesthetic plan, procedure, and co-administered drugs. Evidence identified in the targeted search does not support age-based dose escalation, routine conversion between intranasal and intravenous doses, or use of an injection-pain ED50 as a threshold for analgesia or emergence prophylaxis. Failure to identify an upper boundary does not show that doses above 0.5 mg·kg^−1^ are safe. Dexmedetomidine–esketamine premedication may be considered when sedation, parental separation, analgesia, and ED prevention are simultaneous goals, but it should not yet be described as synergistic or uniformly superior to dexmedetomidine alone. Local regulatory approval, institutional policies, and monitoring standards remain relevant because paediatric indications may be off-label.

### 8.3 Research priorities

Future trials should compare prespecified doses within the same procedure and anaesthetic protocol and randomise timing independently of dose. ED, EA, pain, extubation time, post-anaesthesia care unit stay, and adverse effects should be reported separately. Each study should retain the outcome terminology and validated threshold used by its instrument rather than treating “ED/EA” as a standardised composite endpoint. Studies should prespecify PAED or Watcha thresholds, assessment times, and the handling of pain and residual sedation. Paediatric population PK models should incorporate weight, maturation, route, timing, co-medication, and active metabolite exposure and link these variables to both efficacy and safety. Longer follow-up is needed because current behavioural assessment rarely extends beyond 30 days (5).

### 8.4 Limitations of this review

This mini review used a targeted PubMed/MEDLINE search rather than a systematic multi-database strategy. It did not search trial registries, Embase, the Cochrane Library, Web of Science, Scopus, or non-English databases. We excluded unpublished studies and ongoing trials without peer-reviewed results. Although two authors screened records independently, we did not conduct a formal risk-of-bias assessment, evaluate publication bias, apply GRADE, or perform a quantitative synthesis. Relevant studies may therefore have been missed, and the certainty of the findings cannot be graded. The evidence is also constrained by heterogeneous endpoints, predominantly single-centre studies, limited direct dose comparisons, and the absence of validated paediatric esketamine PK/PD models. Several dose estimates concern immediate procedural responses and should not be extrapolated to sustained analgesia, sedation, or neurodevelopmental safety.

## 9 Conclusion

Among studies identified in the targeted search, 0.2–0.5 mg·kg^−1^ was the best-studied intravenous range for prevention of emergence outcomes in selected surgical settings, especially adenotonsillectomy under sevoflurane anaesthesia. The evidence identified does not establish a fixed therapeutic window and should not be extrapolated to analgesia, procedural sedation, intranasal dosing, or other procedures. Differences across studies are more plausibly explained by the interaction of dose, timing, route, procedure, anaesthetic technique, developmental stage, co-medication, and outcome definition than by a proven reversal at a single threshold. Direct dose–timing comparisons, paediatric exposure–response modelling, endpoint-specific assessment, and longer safety follow-up are required before indication-specific boundaries can be defined.
