# Supplementary material for: Dose- and endpoint-dependent effects of perioperative esketamine in children: evidence from a targeted review
Source: Front Pediatr. 2026 Oct 21;14:1963881. doi: 10.3389/fped.2026.1963881 (PMC13733043; doi:10.3389/fped.2026.1963881)
Supplement: Supplementary file 1 [file Table1.docx]

**Supplementary Table 1. PubMed/MEDLINE search strategy**

| **Database** | PubMed/MEDLINE |
| --- | --- |
| **Search date** | 10 August 2026 |
| **Date coverage** | 1 January 2006 to 10 August 2026 |
| **Fields** | Title/Abstract |
| **Complete search string** | (("esketamine"[Title/Abstract] OR "S-ketamine"[Title/Abstract]) AND ("child"[Title/Abstract] OR "children"[Title/Abstract] OR "paediatric"[Title/Abstract] OR "pediatric"[Title/Abstract] OR "adolescent"[Title/Abstract] OR "adolescents"[Title/Abstract]) AND ("anaesthesia"[Title/Abstract] OR "anesthesia"[Title/Abstract] OR "analgesia"[Title/Abstract] OR "sedation"[Title/Abstract] OR "emergence delirium"[Title/Abstract] OR "emergence agitation"[Title/Abstract] OR "propofol injection pain"[Title/Abstract] OR "dose"[Title/Abstract] OR "dose response"[Title/Abstract] OR "dose-response"[Title/Abstract] OR "pharmacokinetics"[Title/Abstract] OR "pharmacodynamics"[Title/Abstract] OR "intranasal"[Title/Abstract] OR "dexmedetomidine"[Title/Abstract] OR "safety"[Title/Abstract] OR "adverse event"[Title/Abstract] OR "adverse events"[Title/Abstract] OR "haemodynamic"[Title/Abstract] OR "hemodynamic"[Title/Abstract] OR "respiratory"[Title/Abstract] OR "recovery"[Title/Abstract])) AND ("2006/01/01"[Date - Publication] : "2026/08/10"[Date - Publication]) |
| **Records identified** | 166 |
| **Selection** | Title and abstract screening followed by reference-list checking |
| **Included in focused narrative synthesis** | 15 paediatric original studies and 5 directly relevant systematic reviews or meta-analyses |
| **Screening process** | Two authors independently screened the records; disagreements were resolved through discussion |
| **Additional evidence** | Earlier foundational pharmacology, validated outcome instruments, adult pharmacokinetic studies, and animal safety studies were retained only when needed to interpret gaps in direct paediatric evidence. |

Note: The search was designed for a focused Mini Review and was not intended to satisfy systematic-review methodology. The final selection and all reported counts should be confirmed by the authors before submission.
